# Chronic pelvic insufficiency fractures and their treatment

**DOI:** 10.1007/s00402-024-05717-4

**Published:** 2024-12-21

**Authors:** Jan Gewiess, Christoph Emanuel Albers, Marius Johann Baptist Keel, Frede Frihagen, Pol Maria Rommens, Johannes Dominik Bastian

**Affiliations:** 1 Department of Orthopaedic Surgery and Traumatology, Inselspital, Bern University Hospital, University of Bern, Bern, Switzerland; 2 Spine-pelvis AG, Medical School, University of Zurich, Trauma Center Hirslanden, Clinic Hirslanden, Witellikerstrasse 40, CH-8032 Zurich, Switzerland; 3 Department of Orthopaedic Surgery, Østfold Hospital Trust, Grålum, Norway; 4 Institute of Clinical Medicine, University of Oslo, Oslo, Norway; 5 Department of Orthopaedics and Traumatology, University Medical Center Mainz, Mainz, Germany

**Keywords:** Fragility fracture, Pelvic fracture, Osteoporosis, Sacral fracture

## Abstract

Fragility and insufficiency fractures of the pelvis (FFP) and sacrum (SIF) are increasingly prevalent, particularly among the elderly, due to weakened bone structure and low-energy trauma. Chronic instability from these fractures causes persistent pain, limited mobility, and significant reductions in quality of life. Hospitalization is often required, with substantial risks of loss of independence (64–89%) and high mortality rates (13–27%). While conservative treatment is possible, surgical intervention is preferred for unstable or progressive fractures. FFP and SIF are primarily associated with osteoporosis, with 71% of patients not receiving adequate secondary fracture prevention. Imaging modalities play a crucial role in diagnosis. Conventional radiography often misses sacral fractures, while computed tomography (CT) is the gold standard for evaluating fracture morphology. Magnetic resonance imaging (MRI) offers the highest sensitivity (99%), essential for detecting complex fractures and assessing bone edema. Advanced techniques like dual-energy CT and SPECT/CT provide further diagnostic value. Rommens and Hofmann’s classification system categorizes FFP based on anterior and posterior pelvic ring involvement, guiding treatment strategies. Progression from stable fractures (FFP I–II) to highly unstable patterns (FFP IV) is common and influenced by factors like pelvic morphology, bone density, and sarcopenia. Treatment varies based on fracture type and stability. Non-displaced posterior fractures can be managed with sacroplasty or screw fixation, while displaced or unstable patterns often require more invasive methods, such as triangular lumbopelvic fixation or transsacral bar osteosynthesis. Sacroplasty provides significant pain relief but has limited stabilizing capacity, while screw augmentation with polymethylmethacrylate improves fixation in osteoporotic bones. Anterior ring fractures may be treated with retrograde transpubic screws or symphyseal plating, with biomechanical stability and long-term outcomes depending on fixation techniques. FFP and SIF management requires a multidisciplinary approach to ensure stability, pain relief, and functional recovery, emphasizing early diagnosis, tailored surgical strategies, and secondary prevention of osteoporotic fractures.

## Introduction

Fragility fractures of the pelvis (FFP) and sacrum (SIF) from low-energy trauma and atraumatic insufficiency fractures in the elderly are increasing [1, 2]. Chronic instability in these fractures leads to persistent pain, limited daily activity, and reduced ability to return to pre-injury levels [3]. Many cases require hospitalization, with significant loss of independence (64–89%) and high mortality rates (13–27%) [4–9]. Surgical intervention is often recommended over conservative treatment when chronicity is present.

## Etiology and epidemiology

Fragility and insufficiency fractures of the pelvis (FFP) and sacrum (SIF) occur in weakened bones, with fragility fractures caused by forces that deform the bone, while insufficiency fractures result from the bone structure failing without trauma [10]. Insufficiency fractures are more akin to a collapse than the explosive nature of fractures caused by high-energy trauma [11]. FFP and SIF often occur due to low-energy incidents, like falls from standing or sitting, and are particularly prevalent among individuals over 60 years old [12, 13].

Chronicity in FFP/SIF is linked to unstable fracture patterns where abnormal movement occurs under normal loads, preventing proper bone healing. Additionally, fractures that progress over time, with or without increasing instability, lead to long-lasting pain, severe limitations in daily activities, and reduced ability to return to pre-injury levels of activity [1–3]. Even when FFP are recognized and treated correctly, only 20% of patients receive follow-up diagnostics like DXA measurements, leading to a significant lack of secondary fracture prevention [14]. This contributes to the 71% osteoporosis treatment gap in Europe, where the majority of pelvic fractures are related to osteoporosis [15].

Over the past thirty years, there has been a notable rise in the incidence of FFP among older individuals, with a concurrent trend towards more severe lesions and an increase in surgical treatments [1, 16]. Given the risks associated with chronicity, surgical intervention is often recommended over conservative treatment, with a variety of techniques available based on the severity of the condition.

## Clinical presentation

Patients with fragility fractures of the pelvis (FFP) often experience pain in the anterior (symphysis) or posterior (sacrum) pelvic structures, hindering mobility. Sacroiliac joint pain suggests posterior ring involvement (sensitivity 89%, specificity 61%) [17]. Low back pain, common in FFP, may mislead initial evaluation [10]. Neurological impairment, hemorrhage and hemodynamic instability are rare [18]. Delayed diagnosis, often due to difficulty recalling trauma, is linked to more severe fractures and progression, which could be mitigated with early treatment [19, 20].

## Imaging

### Radiography

Proper imaging is crucial for evaluating FFP. Initial studies should include anteroposterior, inlet, and outlet radiographs. The anteroposterior view offers a broad overview, but radiographs have a low sensitivity of 2% for detecting sacral U-type fractures [21]. The inlet view is effective for assessing pelvic ring symmetry and fracture displacement, while the outlet view evaluates vertical sacral fractures. Despite a low sensitivity of 15–48%, conventional imaging is essential for initial workup and follow-up, though reliance on it alone risks missing FFP [22–25].

### Computed tomography

CT imaging is the gold standard for detecting FFP due to its reliability and speed. It can identify additional posterior involvement in 54–97% of cases where anterior lesions are detected via conventional imaging [11, 17, 26, 27]. However, CT alone detects 53–94% of FFP cases [28–30]. Dual-energy CT (DECT) visualizes bone edema, with sensitivity and specificity comparable to MRI [31, 32]. CT is recommended for confirming FFP and evaluating fracture stability, often leading to surgical intervention in 30% of cases [26, 33, 34].

### Magnetic resonance imaging

MRI demonstrates a 99% sensitivity for detecting FFP, making it the most sensitive imaging modality, particularly with fat-suppressed T2-weighted sequences [35]. Studies reveal that 65% of sacral fracture complexity is underestimated by CT alone [29, 30]. MRI is also useful for identifying additional lumbar spine pathologies in 10–50% of cases and distinguishing fractures from metastatic disease using specific signal characteristics [36]. Experts recommend MRI, especially with coronal STIR sequences, for older adults with sudden low back pain and suspected sacral insufficiency fractures (SIF) [30, 37].

### Spect/scintigraphy and pet

**Fig. 1:**
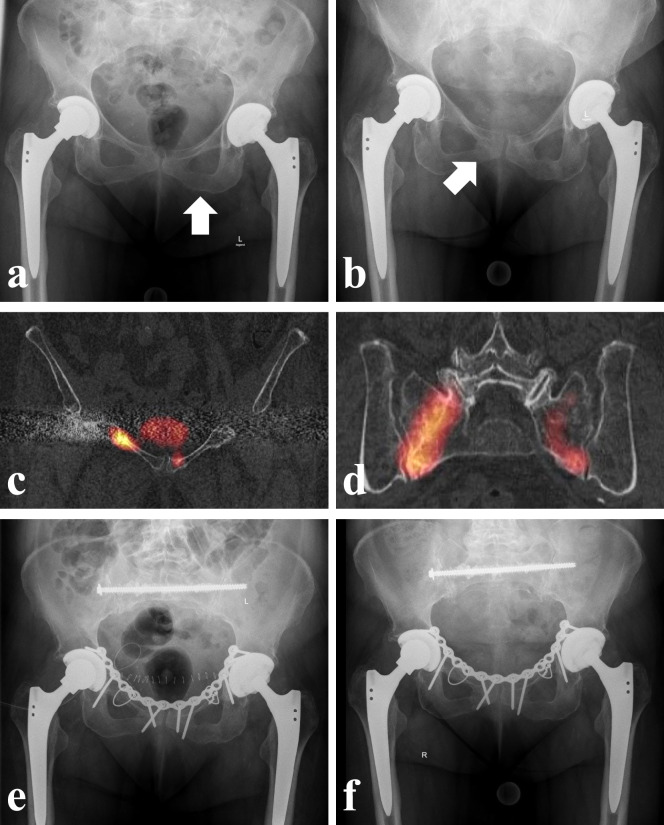
77-year-old female after lateral fall showed in ap pelvis (**a**) a non-displaced pubic ramus fracture on the left side. Conservative treatment with 6 weeks partial weight bearing on the left side was initiated. 6 months later, the patient suffered a new non-displaced pubic ramus fracture on the right side with associated bilateral sacral insufficiency fractures as sign of the crescendo effect of osteoporotic pelvic fractures. Ap pelvis (**b**) and coronal reconstructions of SPECT-computed tomography of the anterior pelvic ring (**c**) and the posterior pelvic ring (**d**). The anterior pelvis was fixed with a reconstruction plate with additional bilateral cerclages around the pubic rami to increase the stability and prevent loosening of the screws. The posterior lesions were stabilized with a cement-augmented transsacral screw (ap pelvis postoperatively (**e**) and 1 year postoperatively (**f**)

Scintigraphy shows high sensitivity for detecting FFP, with symmetric increased radionuclide uptake indicating insufficiency fractures (Fig. 1) [38, 39]. SPECT/CT with 99mTc-MDP provides detailed fracture morphology [40]. In radiotherapy patients, 18 F-FDG PET/CT or PET/MRI offers additional benefits, though availability and radiation protection are challenges [41, 42].

## Comprehensive classification of FFP according to Rommens and Hofmann

Rommens and Hofmann’s comprehensive classification of fragility fractures of the pelvic ring [11] divides isolated anterior fractures (FFP I; a = unilateral, b = bilateral) from non-displaced posterior injuries (FFP II; a = isolated non-displaced sacral fracture without involvement of the anterior ring, b = non-displaced sacral crush with anterior disruption, c = non-displaced sacral, iliosacral or ilium fracture with anterior disruption), displaced unilateral posterior injuries (FFP III; a = displaced unilateral iliac fracture, b = displaced unilateral iliosacral disruption, c = displaced unilateral sacral fracture), and displaced bilateral posterior injury (FFP IV, a = bilateral iliac fracture or bilateral iliosacral disruption, b = bilateral sacral fracture and spinopelvic dissociation, c = combination of different dorsal instabilities). Pieroh et al. reported moderate and substantial intra-rater and inter-rater reliabilities for the FFP classification. They emphasized the importance and challenges of distinguishing between fracture types II and III, as this differentiation influences the decision for nonoperative or operative treatment [43].

## Fracture progression potential

### Epidemiology

Fracture progression in osteopenic pelvic ring injuries is a recognized but under-researched phenomenon [44, 45]. In a study by Rommens et al., there were 21 fracture progressions (FP) in 148 FFP cases, which makes 14,2% [20]. Remarkably, fracture progression occurred in 20 of 111 conservatively treated FFP (18.0%) and in only 1 of 37 surgically treated FFP (2, 7%). This data supports the hypothesis that FP is minimized by operative stabilization. Notably, 40% of women with prolonged pain or restricted mobility experienced progression, leading to ‘crescendo’ instability in 60% of cases (Fig. 1).

### Biomechanical considerations

Biomechanical studies show that a healthy pelvis can withstand up to 6000 N of vertical load before failing [46]. Bone density varies across the anterior pelvic ring, with the highest density in the superior pubic ramus and cotyloid fossa, and lower densities in the inferior pubic ramus [47]. Isolated anterior fractures (FFP I) do not significantly alter pelvic biomechanics, but these fractures often progress, particularly in younger individuals and females [20, 48]. Finite element analysis indicates that superior ramus fractures increase stress on the lower ramus and posterior structures, while complete anterior rami disruption redirects load to the posterior pelvis, significantly increasing stress [49]. Such progression commonly leads to combined anteroposterior disruptions (FFP IIb-c or FFP IIIb-c), reducing stability by at least 30% [20, 50].

Posterior pelvic ring injuries, typically found in the sacral ala, depend on overall bone density, with stress concentrations often aligning with regions of low bone mass [51–53]. Studies reveal fracture progression in 35% of cases with nondisplaced posterior lesions (FFP II), often progressing to FFP III, and ultimately to spinopelvic dissociation (FFP IVb) in nearly half of the patients [20]. Fracture progression starts with unilateral sacral fracture, potentially leading to contralateral involvement and sacral alar collapse, which may result in spinopelvic dissociation [54].

Pelvic morphology and spinopelvic alignment, including sacral slope and pelvic incidence, may influence fracture progression, though their exact role remains controversial [20, 55]. Sarcopenia, while increasingly recognized to compromise clinical outcome in orthopedic surgery [56], has demonstrated an inverse relationship with FFP displacement suggesting that older adults with better muscle mass may experience more severe displacements [57]. However, the link between higher pelvic incidence and fracture progression has shown inconsistent results [20].

## Treatment of fragility fractures of the posterior pelvic ring

Nondisplaced posterior fractures (FFP II) can be treated with sacroplasty, percutaneous SI screws, or bridging plate osteosynthesis [59–62]. Displaced or unstable fractures (FFP III and IV) typically require osteosynthesis, often involving fracture reduction, possibly via an open approach, with fixation methods like those used in FFP II [11, 62–65]. In severe cases with higher degree of instability, especially for FFP IV, complex approaches, including triangular spinopelvic fixation, may be necessary [63].

### Sacroplasty

Sacroplasty is a percutaneous procedure introduced in 2002 that involves injecting polymethylmethacrylate (PMMA) into sacral fracture clefts to stabilize the fractured sacrum and alleviate pain by preventing micromotions [66]. Despite its goal of pain relief, concerns have arisen about its impact on natural bone healing and fracture union, leading some researchers to question its suitability for stabilization of posterior pelvic ring fractures [63, 67].

A multicenter study of 243 patients with osteoporotic sacral insufficiency fractures or pathologic sacral lesions found sacroplasty to be effective and safe, with the Visual Analog Scale (VAS) score dropping from 9.2 ± 1.1 to 1.9 ± 1.7 after up to 1 month [68]. Frey et al. also reported significant pain reduction, with the VAS score decreasing from 8.1 preoperatively to 0.8 at 52 months [69].

Cement leakage is a common complication, potentially affecting veins, neuroforamina, and the intervertebral disc space [60]. However, symptomatic leakage is rare, with isolated cases of radiculitis reported [60, 68, 69]. Mahmood et al. found no major complications in their systematic review, supporting sacroplasty’s safety and effectiveness [70]. Balloon assistance may help prevent cement leakage [71].

New sacroplasty techniques, including the ‘XX’ technique, electromagnetic navigation, and augmented reality, have been proposed but remain unproven [73–74]. Traditional methods involve various needle placements to optimize cement delivery and fracture stabilization [66, 75].

### Screw osteosynthesis of the posterior pelvis (sacroiliac and transsacral screws)

Sacroiliac (SI) and transsacral screw osteosynthesis are percutaneous techniques used for non-displaced (FFP II) and slightly displaced (FFP III and IV) posterior Denis zone 1 and 2 lesions. These procedures are advantageous because they can be performed with the patient in the supine position, simplifying anesthesia management. However, for displaced fractures, obese patients, or aberrant corridors, the prone position is recommended.

Introduced by Chip Routt in 1993, percutaneous SI screw placement requires careful assessment of the screw corridor using sacral foramina on the outlet projection, spinal canal and S1 body on the inlet projection, and iliac cortical densities on the true lateral projection to define a safe zone for screw insertion [77–78]. Pelvises with dysmorphic sacra may present challenges, identified through various imaging projections like outlet views (sacrum not recessed, presence of mamillary processes, dysmorphic S1 foramen), inlet views (anterior cortical indentation), lateral projections (acute sacral slope), and axial CT images (extensive sacroiliac joint interdigitation) [79]. The “minority phenotype” lacks sufficiently large transsacral corridors at S1 or S2 [80]. For dysmorphic sacra, screws should be oriented perpendicular to vertical fracture lines or the vestibule plane, with entry points often more dorsal and distal [81]. The transsacral corridor has varying bone mass distributions, with higher values in S1 compared to S2, and greater mass at the auricular surface cortices and sacral bodies [51].

The pull-out strength of SI screws correlates with screw length and thread length, with screws reaching the opposite sacroiliac joint providing more stability than bilateral SI screws [82, 83]. The breakaway torque is highest in the upper S1 sacral body, with lower values at the sacral ala [51, 52]. The effectiveness of bilateral SI screws versus transsacral fixation remains debated. A second ipsilateral SI screw increases rotation stiffness and load to failure, but combining transsacral and SI screws may be more effective than multiple SI screws alone [85–86]. Recent studies suggest that transsacral screws stabilizing both S1 and S2 reduce the need for additional surgeries [87]. Transsacral fixation offers advantages like better craniocaudal force distribution and increased screw thread purchase [88].

In most cases, slight compression results in sufficient closure of the fracture areas [89]. Bone grafts can enhance stability and promote fusion in SI and transsacral screw osteosynthesis. However, autologous bone grafts from the iliac crest are rarely needed and should be indicated with great caution as it involves a more aggressive, open surgery [90]. Alternatives like allografts and synthetic bone graft substitutes (calcium phosphate ceramics, bioactive glass) show promise but need further investigation [64, 92–93].

Osteoporotic bones may have insufficient screw purchase, making cement augmentation a recommended technique. Cement augmentation increases anchorage and reduces displacement compared to nonaugmented screws and significantly reduces cut-out distance [62, 91, 92]. However, risks include improper cement application leading to leakage. Recent studies suggest that augmenting SI screws at the lateral mass is more effective than at the center of the S1 vertebral body [62]. In U-type FFP fractures, one augmented transsacral S1 screw provides sufficient stability, similar to other techniques (Fig. 1) [64, 65]. Bilateral SI screws showed better rotational stability in finite element analyses, though their clinical advantage remains uncertain [93, 94].

Studies on percutaneous screw fixation in high-energy trauma cases generally affirm its safety [95–98]. Eckardt et al. found positive outcomes in older adults using CT-guided posterior SI and/or transsacral screw implantation but noted a 10% mortality rate, 20% reoperation rate, and 12% loss of independence [87]. Hopf et al. reported significant pain reduction in older adults one week after SI screw fixation, though their study lacked a control group [99].

Implant loosening is a common complication after SI screw fixation, often requiring additional surgery. Causes include rotational moments causing screw rotation within cancellous bone and general implant failure [87, 88]. PMMA augmentation and a combination of SI and/or transsacral screws may help prevent these issues [62].

Malpositioning of SI and transsacral screws, due to the proximity of lumbar and sacral nerve roots, is a rare but serious complication. CT scans of 100 pelvises revealed insufficient corridor dimensions for 7 mm screws in many cases, which supports the need of meticulous preoperative planning [78]. Obesity is a significant risk factor for malpositioning due to challenges in screw placement and visualization during surgery. CT navigation can help minimize these risks [100].

### Transsacral bar osteosynthesis

Sacral bar osteosynthesis is an alternative to posterior screw fixation for FFP II-IV fractures. This technique uses washers and nuts to achieve fracture compression, reducing risks of pull-out and loosening, and is less dependent on bone quality [59]. Initially, Marvin Tile recommended the ilio-iliac approach for younger patients with traumatic injuries [101], but it has been largely abandoned due to issues like loss of reduction and implant complications [102, 103]. In osteoporotic cases, transsacral bar placement can provide adequate interfragmentary compression, provided there is sufficient space for a 6 mm bar [104].

Transsacral bar placement can be challenging due to sacral shape and narrow pathways in S1 and S2. For fractures below S1, such as U-/H-type fractures, additional fixation like sacroiliac screws may be necessary [59]. Recent studies have shown positive outcomes with transsacral bar osteosynthesis. Rommens et al. found comparable physical and mental health scores in 64 patients one-year post-surgery [105], though half required additional sacroiliac screws. Wagner et al. reported a 5% bar loosening rate in 85 patients, with 61% needing additional fixation; 85% were living at home and 82% were walking at three years [59]. Mehling et al. found bony healing in all eleven patients studied, with no revisions [104].

### Posterior plate osteosynthesis

Posterior tension or bridging plate osteosynthesis is recommended for sacral insufficiency fractures, both unilateral and bilateral. Used for three decades in traumatic pelvic injuries [106, 107], this technique involves contouring the plate around the posterior iliac crests or positioning it submuscularly. Compared to sacroiliac (SI) and transsacral screw osteosynthesis, it requires prone positioning, longer incisions, and adequate soft tissue coverage. Kobbe et al. reported that 67% of patients achieved good to excellent outcomes with an average follow-up of 30 months, with only one deep wound infection and no nerve or blood vessel complications [108]. The average surgery duration was 101 min.

### Bilateral (triangular) lumbopelvic fixation

**Fig. 2:**
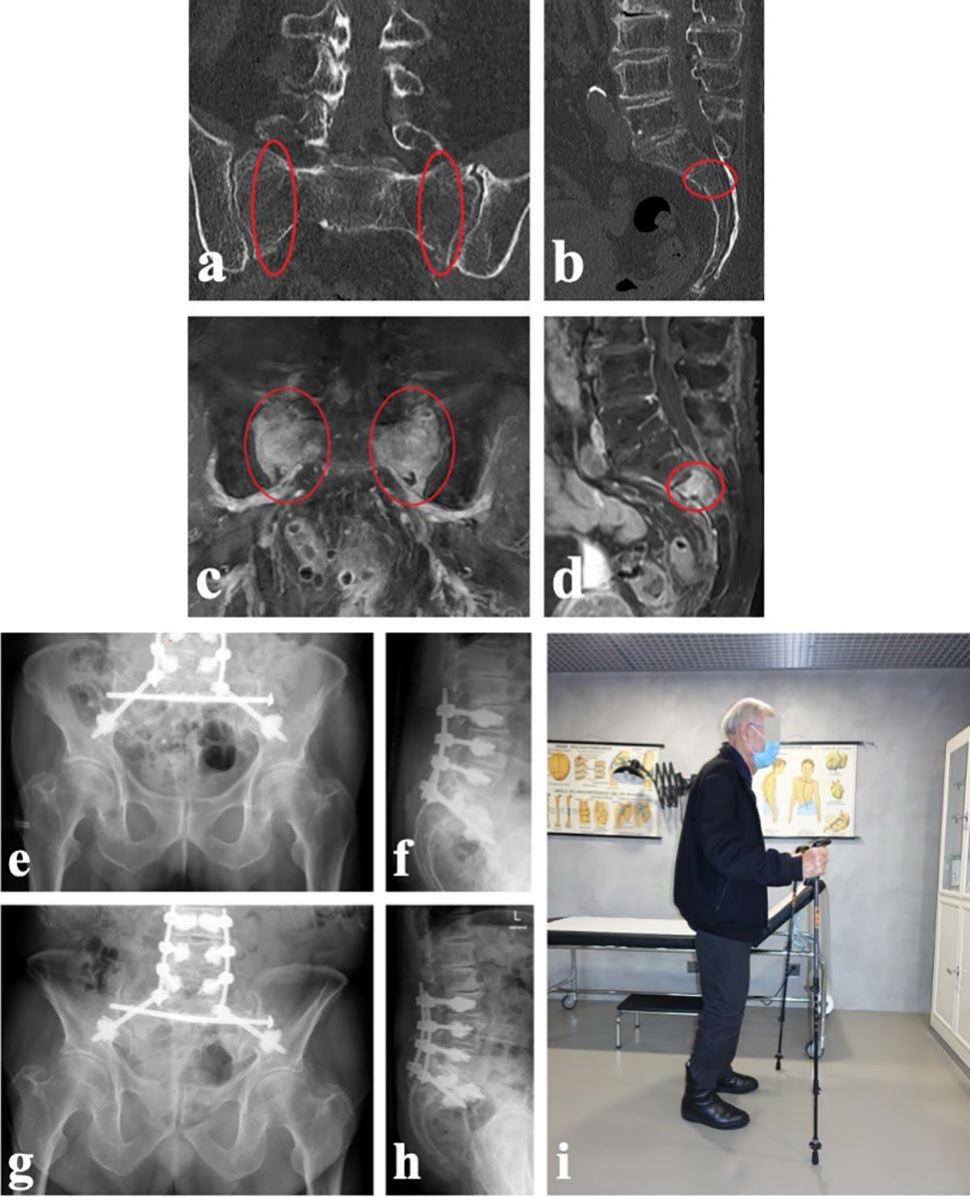
87-year-old male developed immobilizing low back pain due to multilevel lumbar degenerations and a non-displaced H-shaped sacral insufficiency fracture (FFP type IVb—spinopelvic dissociation). Coronal and sagittal reconstructions of computed tomography (**a**, **b**) and magnetic resonance imaging (**c**, **d**) (red circles show the fractures). Postoperative ap pelvis (**e**) and lateral radiograph of the lumbar spine (**f**) and the radiographs one year postoperatively (**g**, **h**) after cement-augmented percutaneous lumbopelvic and transsacral stabilizations with an excellent functional outcome (**i**)

Triangular lumbopelvic fixation is a surgical technique that involves vertical fixation using posterior pedicle screw instrumentation from the lumbar spine to the sacrum, combined with horizontal fixation using sacroiliac (SI) or transsacral screw fixation. This approach is aimed at restoring stability in multiple planes, including the horizontal and vertical planes of the lumbosacral junction [109]. It is typically indicated for displaced and highly unstable fractures, such as spinopelvic dissociations (FFP type IVb - H-/U-type fractures; Fig. 2). The surgical approach for triangular lumbopelvic fixation can be either percutaneous for non- or minimally displaced fractures, or open for cases with displacement, posterior comminution, pseudarthrosis, caudal fractures following instrumented posterior lumbosacral fusion, or neurologic impairment requiring neural decompression [110]. In cases of osteoporotic bone, cement augmentation can be utilized to enhance screw purchase (Fig. 2). The level of lumbar spine pedicle screw instrumentation depends on the fracture pattern. In high-energy trauma, instrumentation from L4 is recommended when there is a fracture located medial to the L5/S1 facet joint to protect this joint. However, in cases of high-grade spinopelvic dissociation or low energy/atraumatic spinopelvic dissociation in older adults with preexisting lumbar degenerative disease, an extension to L4 may not be necessary unless there is involvement of the lumbar spine.

When compared to other posterior stabilization techniques, bilateral triangular lumbopelvic fixation is considered the most stable but also the most invasive method. However, there are no formal indications favoring one technique over the other due to limited evidence on factors such as pain control, early mobilization, procedure-related complications, and mortality rates [111]. Some experts suggest using spinopelvic fixation for displaced fractures and less invasive fixation for nondisplaced fractures [65, 112], while others base their choice on surgeon preference [113]. Lumbopelvic fixation is associated with longer hospital stays, increased operative time, higher implant costs, greater blood loss, and reduced range of motion, but it also increases the likelihood of discharge to home [65, 111, 113, 114]. Its effectiveness has even been questioned in chronic FFP resulting in a crescendo unstable U-type sacral fracture morphology [64]. Recently, Gross et al. proposed a treatment algorithm for FFP IVb type fractures, where the decision to use lumbopelvic fixation depends on the availability of transsacral corridors and the localization of the transverse fracture component [115]. In cases of transverse fractures at the S1/S2 transition zone, lumbopelvic fixation is indicated only when there are no transsacral corridors for at least two single-level.

(S1) transsacral screws. In cases of transverse fractures at the S2/S3 transition zone, lumbopelvic fixation is indicated only when there are no corridors for bilevel (S1 + S2) transsacral screws, two single-level (S1) transsacral screws, or no transsacral corridor at all.

## Treatment of fragility fractures of the anterior pelvic ring

### Retrograde transpubic screw

Retrograde transpubic screw fixation is used for superior pubic ramus fractures, including medial (Nakatani type I), superior (type II), and lateral (type III) to the obturator foramen [116]. It can be applied bilaterally and is performed minimally invasively, leading to minimal blood loss and shorter surgery times. Screws are typically 110–130 mm long, accommodating a 7.3 mm screw [118–119]. They must anchor in the ilium’s lateral cortex above the acetabulum. This technique is suitable for most superior ramus fractures in older adults, as anatomical reduction is often unnecessary.

Biomechanically, retrograde transpubic screw fixation performs comparably to plate fixation in stabilizing the superior pubic ramus in models of sacroiliac screw stabilized FFP IIb fractures and cadaveric APC II type fractures [120]. It improves stability and reduces displacement compared to standalone SI screw fixation [121]. Rommens et al. reported complications in 10% of patients, including screw displacement and local issues [122]. Despite some loss of reduction, the clinical impact is minimal, with a 2% revision rate due to hardware issues [116].

### Symphyseal plating

In symphyseal disruptions, far medial fractures, and parasymphyseal defects, symphysis-spanning plate osteosynthesis is usually adequate [123]. Chronic FFP can lead to symphyseal instability from bone resorption due to repetitive movement (Fig. 1). Severe displacement often requires plating because of better fracture visualization and reduction compared to minimally invasive techniques. Longer plates or double plate osteosynthesis can improve fixation strength over transpubic screw methods. Herteleer et al. found that double plating resulted in less frequent and less severe screw loosening, with revision needed only for single plate cases [124].

## Conclusion

In conclusion, fragility fractures of the pelvis (FFP) and sacrum insufficiency fractures (SIF) are increasingly common, especially in older adults, leading to significant morbidity, loss of independence, and even mortality. Chronicity in these fractures, often stemming from delayed or improper diagnosis, exacerbates the condition, causing severe pain and limited mobility. While conservative treatment may suffice in non-displaced fractures, surgical interventions are increasingly favored, particularly for unstable fractures, to prevent progression and ensure better outcomes. Advances in imaging techniques, such as CT and MRI, have improved the diagnosis and treatment planning of FFP and SIF. Moreover, innovative surgical techniques, including sacroplasty, screw osteosynthesis, and lumbopelvic fixation, offer effective stabilization options. However, the choice of treatment must be tailored to the patient’s fracture type, bone quality, and overall health, with surgery often preferred to mitigate risks of fracture progression and to enhance recovery and mobility. Despite these advancements, there remains a need for further research into long-term outcomes and optimal treatment strategies for osteoporotic fractures.

## Data Availability

No datasets were generated or analysed during the current study.
